# Modular Development of a *Klebsiella pneumoniae* Bioconjugate Nanovaccine Elicits Robust Protection via Intranasal Immunization

**DOI:** 10.3390/nano16070428

**Published:** 2026-03-31

**Authors:** Zhenshi Li, Lingli Chen, Canran Liu, Kangfeng Wang, Juntao Li, Xue Yan, Yuqing Jiang, Yan Guo, Li Zhu, Hengliang Wang, Chao Pan

**Affiliations:** 1 College of Food Science and Technology, Shanghai Ocean University, Shanghai 201306, China; 18716532473@163.com; 2 National Key Laboratory of Advanced Biotechnology, Academy of Military Medical Science, Beijing 100071, China; chenlinglizy@163.com (L.C.); 18772103166@163.com (C.L.); wangkf1220@126.com (K.W.); ljtanlz0046@163.com (J.L.); cynthia2182@163.com (X.Y.); jyq1214179706@163.com (Y.J.); yanguobrilliant@163.com (Y.G.); jewly54@bmi.ac.cn (L.Z.); 3 School of Basic Medical Sciences, Fujian Medical University, Fuzhou 350108, China; 4 State Key Laboratory of Pathogen and Biosecurity, Academy of Military Medical Sciences, Beijing 100071, China

**Keywords:** *Klebsiella pneumoniae*, nanovaccine, modular assembly, intranasal immunization, glycosylation

## Abstract

*Klebsiella pneumoniae* poses a severe global health threat due to its extensive antibiotic resistance. However, to date, no vaccine against this pathogen has been approved for clinical use worldwide. Although self-assembling nanocarriers present distinct advantages for vaccine design, their ability to effectively load polysaccharide antigens and further elicit mucosal immunity remains unclear. Here, we developed a modular, self-assembling nanovaccine (CNP-OPS_KpO1_) against *K. pneumoniae* by loading of *K. pneumoniae* O1 polysaccharide antigen onto a cholera toxin B subunit (CTB)-based nanoparticle (CNP). After determining the safety of the vaccine via intranasal immunization, we further evaluated its immune efficacy. CNP-OPS_KpO1_ elicited stronger systemic IgG and mucosal sIgA responses than non-nanoparticulate controls. In a non-lethal pulmonary infection model, CNP-OPS_KpO1_ vaccination reduced lung bacterial burden by over 5 logs compared to controls, achieving near-complete bacterial clearance. Histopathological analysis further confirmed minimal lung damage in vaccinated animals. In addition, in a lethal pulmonary challenge model, it conferred 90% survival, whereas all mice in the antigen-alone control group died within 4 days. Our work not only provides a safe, effective, and adjuvant-free candidate vaccine against *K. pneumoniae* but also advances a versatile platform for developing broad-spectrum mucosal vaccines against other pathogens.

## 1. Introduction

*Klebsiella pneumoniae* is a common Gram-negative opportunistic pathogen widely distributed in both the natural environment and on the mucosal surfaces of the human respiratory and intestinal tracts [1,2]. It can cause a range of severe infections, including pneumonia, bloodstream infections, urinary tract infections, and liver abscesses [3]. Antimicrobial resistance has become a major concern with *K. pneumoniae*, and in recent years, there has been a continuous increase in reports of multidrug-resistant strains, with particular attention to the rising detection of carbapenem-resistant *K. pneumoniae* (CRKP) [4,5]. Vaccines have played a significant role in preventing infectious diseases; however, no licensed vaccine against *K. pneumoniae* is currently available worldwide.

Conjugate vaccines are regarded as the most successful bacterial vaccines currently available [6]. They are produced by coupling highly specific polysaccharides, including capsular polysaccharides (CPS) and O-polysaccharides (OPS) present on the surface of bacteria, with appropriate carrier proteins [7]. When receiving a conjugate vaccine, both T cells and B cells can be stimulated, resulting in long-lasting protection and immune memory [8]. Although currently conjugate vaccines are mainly synthesized by chemical methods, a rapidly advancing biosynthesis method, in recent years, has been widely applied in the development of *K*. *pneumoniae* vaccines. Unlike traditional chemical methods, which are multi-step, time-consuming, and complex, this biotechnological approach requires only the establishment of a glycosylation system in the host to directly produce glycoproteins through a one-step catalytic reaction [9]. In this system, the most important component is glycosyltransferase, primarily including PglB (derived from *Campylobacter jejuni*), PglS (derived from *Acinetobacter baylyi*), and PglL (derived from *Neisseria meningitidis*) [9], which require the ability to recognize exogenous polysaccharides. For *K. pneumoniae*, a quadrivalent conjugate vaccine was developed using the PglS system. Four types of CPSs were respectively coupled with *Pseudomonas aeruginosa* Exotoxin A (EPA), and the vaccine’s immune protection was confirmed in mice [10]. However, the existence of over 100 distinct K. pneumoniae CPS types, with approximately 15–20 types needed to cover more than 70% of infections, makes it challenging to develop multivalent vaccines for broad protection [11,12]. In contrast, OPS exhibits limited serological diversity, with four major serogroups (O1, O2, O3, O5) accounting for more than 80% of clinical isolates, making OPS an attractive target for developing broader-coverage vaccines [13,14,15]. A tetravalent O-antigen bioconjugate vaccine, Kleb4V, developed using the PglB system, has become the first *K. pneumoniae* conjugate vaccine candidate to reach clinical evaluation [16]. Additionally, our team has established a PglL-based system and demonstrated its feasibility in various pathogen models such as *Shigella*, *Salmonella*, and *Acinetobacter baumannii* [17,18,19]. Notably, we found that this system can also recognize *K. pneumoniae* OPS [20], which is advantageous for preparing more effective candidate vaccines.

Advances in nanocarrier-based delivery systems have provided powerful tools for vaccine development [21,22]. Various platforms, including virus-like particles, ferritin nanocages, liposomes, and polymer nanoparticles, have shown great promise in this field [22,23,24]. In our previous work, we developed a modular self-assembling CTBTri nanoparticle (CNP) by fusing a pentameric cholera toxin B subunit (CTB) with a trimeric protein (Tri) [25]. Furthermore, the strong immune activation effect of these CNP-based conjugate vaccines was validated using different pathogen models [26,27,28]. Although CTB modules have been used as clinically validated mucosal adjuvants, suggesting that CNP may be more suitable for inducing mucosal immune responses, these candidate vaccines were administered via injection, and the mucosal immune response—especially against weakly immunogenic polysaccharides—was not evaluated. Additionally, due to the simultaneous occurrence of complex protein self-assembly and glycosylation modification, potential interference between these processes may occur. Therefore, we aim to further explore modular design strategies to achieve efficient loading of polysaccharide antigens onto self-assembled nanocarriers.

In this study, we developed a novel CNP-based nano-conjugate vaccine, CNP-OPS_KpO1_, targeting the *K. pneumoniae* O1 strain, and we evaluated its mucosal immunogenicity and protective efficacy in mice. In our design, the two components—OPS_KpO1_ conjugated to SpyCatcher (SC) (SC-OPS_KpO1_) and CNP containing a SpyTag (ST)—were prepared separately via glycosylation modification and fusion expression. Leveraging the spontaneous isopeptide bond formation between SC and ST, the final vaccine candidate, CNP-OPS_KpO1_, was obtained simply by mixing SC-OPS_KpO1_ with CNP. After characterizing its physicochemical properties and conducting safety assessments, the mucosal immune response and protective efficacy of CNP-OPS_KpO1_ were evaluated in mice. The results demonstrated that intranasal immunization with CNP-OPS_KpO1_ elicited robust humoral and respiratory mucosal immune responses and provided effective protection against both non-lethal and lethal lung infections. To our knowledge, this is the first evaluation of the effect of CTB-based self-assembled nanoparticles on weakly immunogenic polysaccharides in mucosal immunity. This work not only presents a safe, effective, and adjuvant-free candidate vaccine against *K. pneumoniae*, but it also offers a novel strategy for addressing CRKP infections and advances the application of the CNP platform in mucosal vaccine development.

## 2. Materials and Methods

### 2.1. Strains and Plasmids

*Escherichia coli* W3110ΔwaaL, which lacks O-antigen ligase, was constructed and stored in our laboratory [29]. The glycosylation plasmid pET28a-pglL-SC4573 (co-expressing glycosyltransferase and SC4573) and the *K. pneumoniae* O1 polysaccharide synthesis plasmid PACYC184-KPO1 were also constructed in our lab [29]. These two plasmids were sequentially transformed into *E. coli* W3110ΔwaaL cells to generate the recombinant strain W3110ΔwaaL/pET28a-pglL-SC4573, PACYC184-KPO1. The wild-type *K. pneumoniae* strain 041 (serotype O1) was stored in our laboratory.

### 2.2. Bacterial Culture and Protein Expression

Strains were inoculated into LB medium supplemented with the appropriate antibiotics (50 µg/mL kanamycin and 50 µg/mL chloramphenicol as required) and cultured overnight at 37 °C with shaking at 220 rpm. The culture was then subcultured (1% *v*/*v*) into fresh LB medium containing antibiotics and grown at 37 °C until the OD600 reached 0.6–0.8. Protein expression was induced by adding 1 mM isopropyl β-D-1-thiogalactopyranoside (IPTG), followed by continued incubation at 30 °C with shaking at 220 rpm for 12–14 h.

### 2.3. Western Blot

The Western blot procedure followed previously described methods [30]. Briefly, samples were mixed with an equal volume of 2× SDS loading buffer and boiled for 10 min. After centrifugation (10,000 rpm, 20 min), the supernatant was loaded onto an SDS-PAGE gel for protein separation. A PVDF membrane was activated by soaking in methanol followed by transfer buffer. Protein transfer was performed using the eBlot™ L1 rapid wet transfer system (GenScript, Nanjing, China). The membrane was then blocked with blocking buffer (TBST containing 5% skim milk) at 37 °C for 2 h. Subsequently, the membrane was incubated with appropriately diluted primary antibodies—either Anti-His-HRP (CWBIO, Cambridge, MA, USA, CW0285M), anti-CTB (Sigma-Aldrich, St. Louis, MO, USA, SAB4200844), or *K. pneumoniae* O1 antiserum—with gentle shaking at room temperature for 1 h. For Anti-His-HRP, after incubation, the membrane was washed three times with TBST (7 min per wash). Protein bands were visualized using a super-sensitive ECL luminescence reagent (EASYBIO, Seoul, Republic of Korea, BE6705-100) according to the manufacturer’s instructions. For anti-CTB or O1 antiserum, following primary antibody incubation and washing (as above), the membrane was incubated with an HRP-conjugated goat anti-mouse (AbMART, Shanghai, China, M21001L) or HRP-conjugated goat anti-rabbit secondary antibody (AbMART, Shanghai, China, M21002L) at 37 °C for 1 h. After washing again, signals were developed and detected using an Imaging System (Tanon-5200, Tanon, Shanghai, China).

### 2.4. TEM

Samples were applied to freshly glow-discharged 200-mesh copper grids and incubated for 5 min. Excess liquid was removed using filter paper, and the grids were stained with 3% (*w*/*v*) uranyl acetate for 45 s. After air-drying, samples were imaged using a JEM-1200EX transmission electron microscope (JEOL, Tokyo, Japan) operated at 100 kV.

### 2.5. Protein Purification

For glycoprotein purification, induced cell pellets were harvested and resuspended in Buffer A1 (20 mM Tris-HCl pH 7, 10 mM imidazole, 500 mM NaCl) at a ratio of 10 mL per gram of wet cell mass. Cells were lysed using a high-pressure homogenizer (PhD Technology LLC, Ames, IA, USA), and the lysate was centrifuged at 8000 rpm for 30 min to collect the supernatant. A HisTrap™ Excel 5 mL column (Sigma Aldritch, St. Louis, MO, USA) was equilibrated with Buffer A1 until stable UV absorbance was achieved. Then, the supernatant was loaded. The column was washed with Buffer A1 to remove nonspecifically bound proteins. Target glycoproteins were eluted with Buffer B1 (20 mM Tris-HCl pH 7, 500 mM imidazole, 500 mM NaCl). The eluted samples were dialyzed against Buffer A (20 mM Tris-HCl pH 7) for about 3 days. The dialysate was then applied to a HiTrap™ Q HP 5 mL column pre-equilibrated with Buffer A (Sigma Aldritch, St. Louis, MO, USA). After loading, the column was washed with Buffer A1 to remove impurities. Bound glycoproteins were eluted using a linear gradient of Buffer B (20 mM Tris-HCl pH 7, 1 M NaCl). Fractions showing increased UV absorbance were collected and analyzed by Coomassie blue staining. The expression and purification of CTBTriST (with SpyTag fused at the C-terminus) were performed as previously reported.

### 2.6. BCA Protein Quantification

Protein concentration was determined using the Micro BCA^TM^ Protein Assay Kit (Thermo Scientific, Waltham, MA, USA, 23235) according to the manufacturer’s instructions. Briefly, a 2 mg/mL BSA standard stock solution was diluted to 100 µg/mL. A standard curve was prepared with BSA concentrations of 0, 5, 10, 20, 50, 60, 80, and 100 µg/mL in a 96-well plate. Test samples were appropriately diluted. BCA working reagent was prepared by mixing Buffer A, Buffer B, and Buffer C at a ratio of 500:480:20. Each well received 100 µL of sample and 100 µL of working reagent. The plate was incubated at 37 °C for 2 h, and absorbance was measured at 562 nm using a microplate reader. Sample concentrations were calculated based on the standard curve.

### 2.7. Polysaccharide Quantification

A 1 mg/mL glucose stock solution was diluted to 100 µg/mL. A standard curve was prepared with glucose concentrations of 0, 5, 10, 20, 50, 60, 80, and 100 µg/mL. Glycoprotein samples were appropriately diluted, and 250 µL of each was aliquoted into tubes. An anthrone–sulfuric acid reagent was prepared by dissolving 2 mg of anthrone per mL of sulfuric acid. One mL of the reagent was added to each tube, followed by immediate cooling in an ice-water bath. After reaching room temperature, tubes were boiled for 10 min to complete the reaction and then cooled again. A 200 µL volume of reacted solution from each tube was transferred to a 96-well plate, and absorbance was measured at 620 nm. Carbohydrate concentration was calculated using the standard curve.

### 2.8. LPS Extraction

Bacteria were inoculated (1:100 *v*/*v*) into 1 L of medium and cultured at 37 °C, 220 rpm for 12–14 h. Bacteria were harvested by centrifugation (8000 rpm, 1 h) and washed three times with pre-cooled ddH_2_O. The pellet was resuspended in ddH_2_O (3 mL per gram wet weight) in a 50 mL centrifuge tube. The suspension was subjected to three cycles of alternating incubation in an ice bath (3 min) and a 68 °C water bath (3 min). An equal volume of 90% phenol was then added, and the mixture was incubated at 68 °C with vigorous shaking for 30 min. After centrifugation at 10,000 rpm for 15 min at 4 °C, the upper aqueous phase was carefully collected. Then, by refilling with ddH_2_O, the mixture was again vigorously agitated as described above and aqueous phase was carefully collected. Combined aqueous phases were dialyzed extensively against ddH_2_O for ≥3 days to remove phenol. The retentate was treated with DNase and RNase at 37 °C for 3 h, followed by incubation with proteinase K at 60 °C for 6 h. Then, the sample was boiled for 10 min, centrifuged (10,000 rpm, 30 min), and the supernatant containing purified LPS was collected.

### 2.9. Glycoprotein Staining

Glycoproteins were stained using the Pierce^TM^ Glycoprotein Staining Kit (Thermo Scientific, Waltham, MA, USA, 24562) per the manufacturer’s protocol. Briefly, samples were separated by SDS-PAGE and the gel was fixed in 100 mL of 50% methanol for 30 min, then washed twice with 100 mL of 3% acetic acid (10 min per wash). The gel was oxidized in 25 mL of oxidation solution for 15 min with shaking, followed by two washes with 3% acetic acid (5 min each). Staining was performed in 25 mL of glycoprotein staining solution for 15 min, after which the gel was treated with 25 mL of reducing solution for 5 min. Finally, the gel was thoroughly washed with 3% acetic acid and then washed with ddH_2_O. Glycoproteins appeared as magenta bands.

### 2.10. Mouse Immunization

Female BALB/c mice (6 weeks old, SPF grade) were purchased from Beijing Vital River Laboratory Animal Technology Co., Ltd. (Beijing, China). All animal experimental procedures were conducted in compliance with the guidelines of the Academy Animal Care and Use Committee (IACUC Approval Code: IACUC-DWZX-2025-050). Mice were randomly assigned to experimental groups. Immunizations were administered intranasally on days 0, 14, and 28. Blood samples were collected via retro-orbital bleeding on days 7, 21, and 35. Serum was separated and stored at −20 °C for subsequent analysis.

### 2.11. Vaccine Safety Evaluation

Mice were immunized with 10 µg of antigen per dose. Post-immunization, mice were placed in metabolic cages (CLAMS, Columbus, OH, USA) to monitor oxygen, carbon dioxideconsumption and heat production. Body temperature and weight were measured before immunization and on days 1, 4, 8, 12, 24, and 30 post-immunization.

Blood samples were collected before and at 12 h, 1, 3, 7, 14, and 30 days after immunization for analysis of serum inflammatory cytokines using corresponding kits (JINGMEI, JM-02446M1, JM-02323M1, JM-02415M1, JM-02465M1). Briefly, The sample was diluted 5 times with sample diluent (total volume 50 μL), and 50 μL of standard was added to each well. The plate was sealed with a membrane and incubated at 37 °C for 30 min. After washing the plate with 1 x washing solution, add 50 μL of horseradish peroxidase (HRP) labeled detection antibody to each well, seal with a plate membrane, and incubate at 37 °C for 30 min. After washing the plate, add 50 μL of substrate A and 50 μL of substrate B to each well, and incubate at 37 °C in the dark for 15 min. Then, add 50 μL of termination solution to each well and measure the absorbance at 450 nm using an enzyme-linked immunosorbent assay reader (Tecan F50, Männedorf, Switzerland).

On day 30, serum biochemical indices—including renal function markers (BUN), muscle enzyme (LDH), and liver function markers (AST, ALP, ALT)—were measured using a biochemical analyzer (Chemray800, Rayto, Shenzhen, China) by Servicebio Biotechnology Co., Ltd. (Beijing, China).

On day 30 post-immunization, heart, liver, spleen, lungs, and kidneys were harvested for histopathological analysis via hematoxylin–eosin (H&E) staining. Organs were initially fixed in 4% paraformaldehyde (Solarbio, Beijing, China), then embedded in paraffin, sectioned, and subjected to H&E staining using a commercial kit (Solarbio) according to the manufacturer’s guidelines. In brief, after embedding and sectioning, the fixed tissues underwent conventional dewaxing and hydration. Subsequently, the sections were stained with H&E, followed by sequential dehydration, clearing, and mounting with neutral resin. Then, the slices were photographed using a white light microscope (SWE-CX63, ServiceBio, Beijing, China).

### 2.12. ELISA

LPS was diluted in coating buffer (50 mM Na_2_CO_3_–NaHCO_3_, pH 9.6) to a concentration of 10 µg per 100 µL and used to coat 96-well plates overnight at 4 °C. Plates were washed with PBST (PBS containing 0.05% Tween-20) using a plate washer, then blocked with 200 µL per well of blocking buffer (5% skim milk in PBST) at 37 °C for 2 h. After washing, serially diluted serum samples (in PBST containing 0.5% skim milk) were added (100 µL/well) and incubated at 37 °C for 1 h. Each plate included negative and blank controls. Then, plates were washed again and incubated with HRP-conjugated secondary antibodies, such as anti-IgG, anti-IgA, anti-IgG1, or anti-IgG2a, and diluted 1:15,000 in dilution buffer (100 µL/well) at 37 °C for 1 h. After a final wash, 100 µL of TMB substrate was added per well. The reaction was stopped with 50 µL of stop solution after 5 min, and absorbance was measured at 450 nm using an enzyme-linked immunosorbent assay reader (Tecan F50, Männedorf, Switzerland).

### 2.13. Scoring Pathological Changes in Lung Slices

Pulmonary pathological changes were evaluated by two experienced pathologists in a blinded manner according to the terminology and scoring criteria established in the International Harmonization of Nomenclature and Diagnostic Criteria for Lesions in Rats and Mice (INHAND). A four-tier grading system was used as follows: 0, within normal range (considering factors such as animal age, gender, and strain, the tissue is considered normal under research conditions); 1, very slight (changes just exceeding the normal range); 2, mild (lesions are observable but not severe); 3, moderate (lesions are obvious and likely more severe); 4, severe (lesions are very severe, affecting the entire tissue and organ).

### 2.14. Statistical Analysis

All data were analyzed using GraphPad Prism 8.4.3 software (GraphPad Inc., San Diego, CA, USA). Parametric tests were used when the data met the assumptions of normality, homogeneity of variance, and independence. Comparisons among multiple groups were performed using one-way ANOVA followed by Dunnett’s multiple comparison test. Comparison between two groups were performed using Student’s *t*-test. Data are presented as mean ± standard deviation. A *p*-value < 0.05 was considered statistically significant (**** *p* < 0.0001, *** *p* < 0.001, ** *p* < 0.01, * *p* < 0.05).

## 3. Results

### 3.1. Preparation and Characterization of Nanoparticle Chassis and K. pneumoniae Antigen for Conjugation

**Figure 1:**
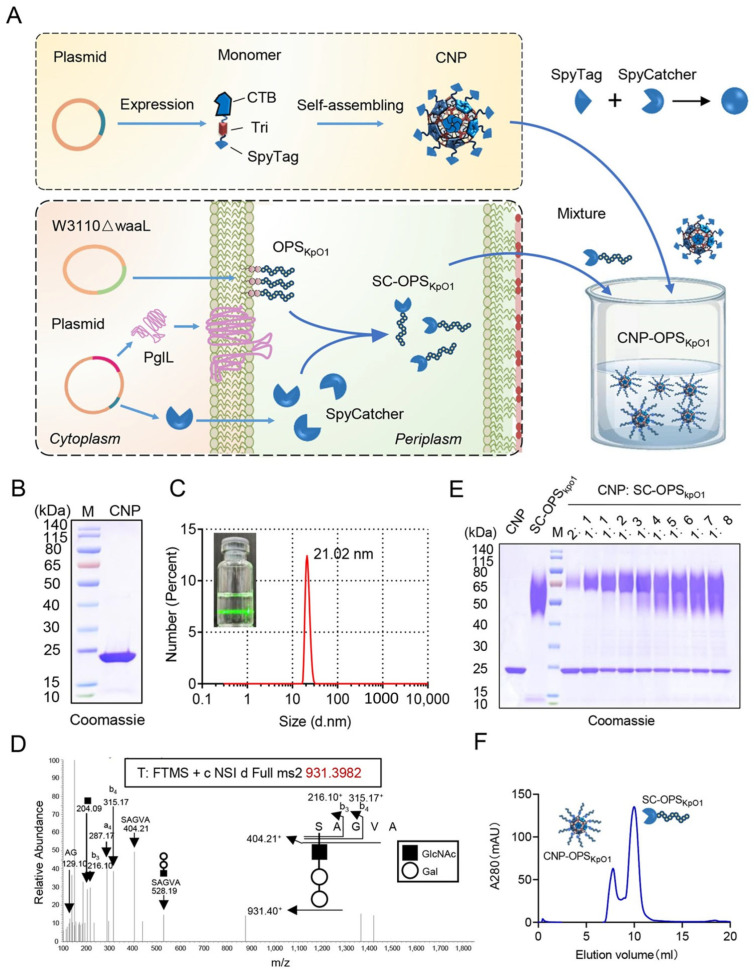
Preparation of CNP-OPS_KpO1_. (**A**) Schematic diagram of CNP-OPS_KpO1_ preparation. (**B**) Coomassie Blue staining of CNP. (**C**) Dynamic light scattering (DLS) and Tyndall effect of CNP. (**D**) LC-MS/MS analysis of SC-OPS_KpO1_. (**E**) Coomassie Blue staining of CNP conjugated with SC-OPS_KpO1_ at different molar ratios. (**F**) Molecular sieve purification of CNP-OPS_KpO1_.

In previous studies, we demonstrated that the fusion expression of CTB and Tri enables self-assembly into nanoparticles, which can subsequently load protein antigens via a biorthogonal system. Given the growing concern over *K. pneumoniae* due to its antibiotic resistance, we aim to leverage this system to develop a candidate vaccine against *K. pneumoniae* using OPS of O1 serotype of *K. pneumoniae* (OPS_KpO1_) as antigen. The nanoparticles chassis CTBTri nanoparticle (CNP) encoding CTBTri with a C-terminal SpyTag (ST) fusion was produced as described before. Then, a plasmid, containing *K. pneumoniae* O1 polysaccharide biosynthesis gene cluster, and a plasmid, expressing both the glycosyltransferase PglL and SpyCatcher (SC) containing the glycosylation modification sequence 4573, were introduced into an engineered *E. coli* W3110 strain. Within this recombinant strain, the synthetic OPS_KpO1_ could be conjugated to SC under the catalysis of PglL, yielding the glycosylated product SpyCatcher-OPS_KpO1_ (SC-OPS_KpO1_). Subsequently, purified SC-OPS_KpO1_ could be loaded on CNP through a simple mixture (Figure 1A). Although SDS-PAGE results indicated that CNP monomer is only about 25 kDa (Figure 1B), in its natural state, it exhibited a pronounced Tyndall effect upon single laser irradiation, suggesting a nanoscale size. Dynamic light scattering (DLS) further demonstrated that it presents as monodisperse particles with a size of approximately 20 nanometers (Figure 1C and Figure S1), which is consistent with previous reports.

To obtain SC-OPS_KpO1_, both *K. pneumoniae* O1 polysaccharide synthesis plasmid PACYC184-KPO1 and glycosylation plasmid pET28a-pglL-SC4573 were transferred into W3110ΔwaaL. After induction with IPTG, SC-OPS_KpO1_ was obtained through affinity chromatography and ion exchange chromatography (Figure S2). The results of Coomassie Blue staining showed that the purity of glycoprotein reached over 90%. To determine the structure of glycan, SC-OPS_KpO1_ strips were cut from SDS-PAGE gel and digested by proteinase K. The glycopeptides obtained were detected by liquid chromatography tandem mass spectrometry (LC-MS/MS). We detected that N-acetyl sugars were connected to serine, followed by two hexose repeat units (Figure 1D), which are consistent with the structure of known *K. pneumoniae* O1 polysaccharides. Then, we mixed the prepared CNP with SC-OPS_KpO1_ in different proportions at 4 °C overnight. After SDS-PAGE separation, the results of Coomassie Blue staining showed that, despite the consistent amount of CNP added, the individual CNP bands gradually weakened with the increase in SC-OPS_KpO1_, and a cluster of new bands appeared at about 65 kDa, with a molecular weight equal to the sum of CNP monomer and SC-OPS_KpO1_ (Figure 1E and Figure S3), indicating successful coupling of CNP with SC-OPS_KpO1_. And from 1:4, the CNP band no longer decreased, indicating saturation of the binding. This result indicates that SC-OPS_KpO1_ can be successfully loaded on the surface of CNP.

After determining that the polysaccharides of *K. pneumoniae* can be loaded onto CNP through a bioorthogonal system, we expanded the binding amount with a slight excess of SC-OPS_KpO1_ and then removed unbound SC-OPS_KpO1_ through molecular sieves. We observed two distinct UV absorption peaks in the chromatogram, with the first peak emerging at approximately 8 mL (about 30% of the column volume), suggesting the presence of aggregated forms (Figure 1F). Subsequent analysis of the two peaks confirmed that, as expected, the first peak corresponded to the conjugated CNP-OPS_KpO1_, while the second peak represented the excess unbound SC-OPS_KpO1_ (Figure 1F and Figure S4).

### 3.2. Characterization of Modular Self-Assembling Nanovaccines Against K. pneumoniae

**Figure 2:**
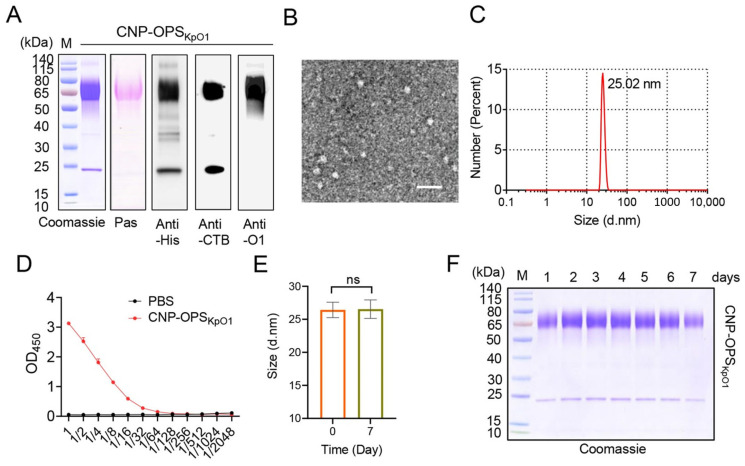
Characterization of CNP-OPS_KpO1_. (**A**): Coomassie Blue staining, glycan staining, and Western blot (WB) analysis of CNP-OPS_KpO1_. (**B**): TEM image of CNP-OPS_KpO1_ (scale bar = 50 nm). (**C**): DLS analysis of CNP-OPS_KpO1_. (**D**): GM1 binding capacity of CNP-OPS_KpO1_. (**E**): Particle size stability analysis of CNP-OPS_KpO1_ via DLS. ns: No significant difference. (**F**): Coomassie Blue staining of CNP-OPS_KpO1_ after different incubation periods at 25 °C.

Upon obtaining CNP-OPS_KpO1_, we performed further characterization. Coomassie Blue staining results of CNP-OPS_KpO1_ show a high purity of over 90%, and polysaccharide staining can detect the presence of polysaccharides at approximately 65kDa (Figure 2A). In addition, the protein and polysaccharide components were identified by WB (anti-His, anti-CTB, and anti-O1 serum), confirming the successful loading of SC-OPS_KpO1_ in the final product (Figure 2A). Afterwards, CNP-OPS_KpO1_ was observed through transmission electron microscopy that it had a rough spherical surface with a size of approximately 25 nm (Figure 2B). The DLS results indicated that CNP-OPS_KpO1_ was slightly larger than CNP due to the loaded OPS_KpO1_, exhibited uniform monodispersity, and its size was consistent with the transmission electron microscopy results (Figure 2C and Figure S5). Furthermore, we tested the GM1 binding ability of CNP-OPS_KpO1_ and the results showed that it maintained the affinity for GM1 (Figure 2D). In addition, CNP-OPS_KpO1_ also demonstrated high stability. After being stored at room temperature (25 °C) for at least 7 days, DLS results showed that its particle size remained consistent with the initial dimensions, with no signs of aggregation or degradation (Figure 2E,F).

### 3.3. Safety Evaluation of CNP-OPS_KpO1_

**Figure 3:**
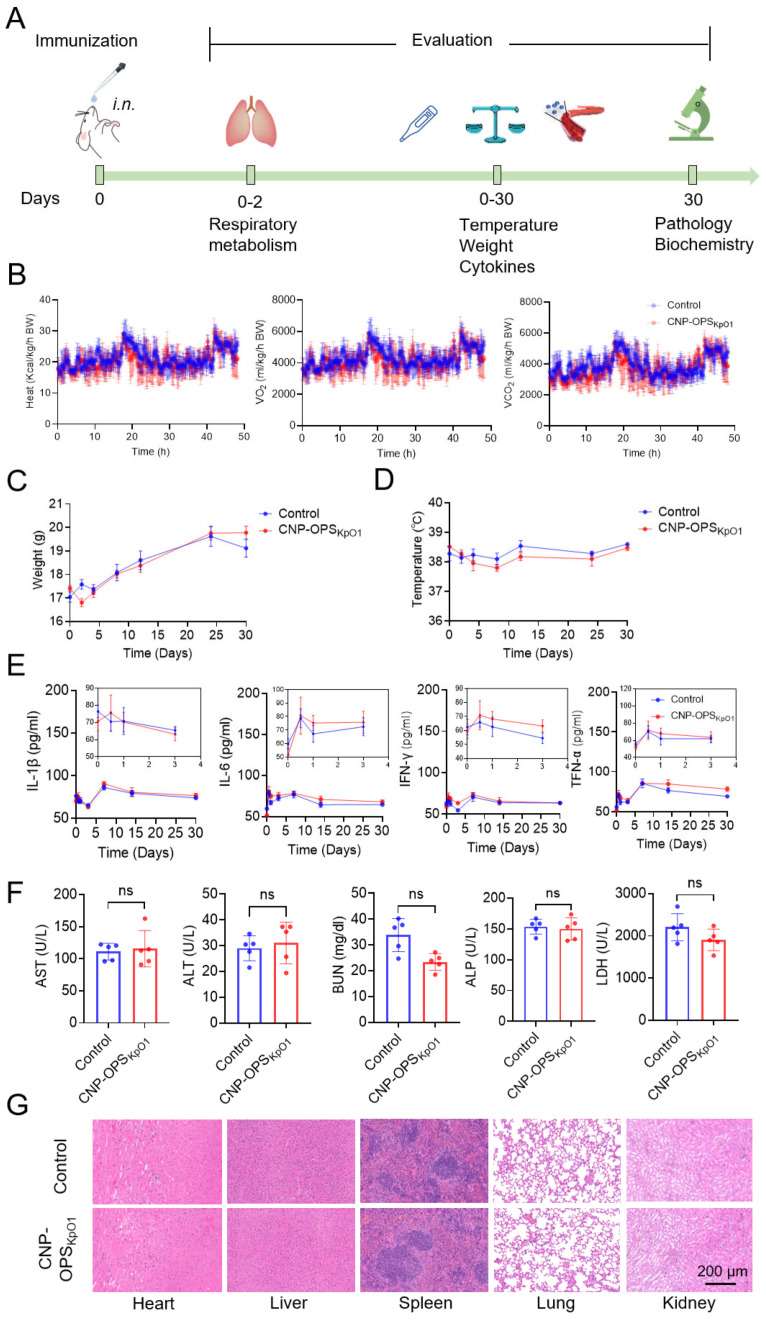
Safety evaluation of CNP-OPS_KpO1_. (**A**): Schematic diagram of the safety evaluation experimental design. (**B**): Heat, VO_2_, and VCO_2_ were measured by metabolic cages (*n* = 4). (**C**,**D**): Body weight and temperature of each mouse were measured during observstion (*n* = 5). (**E**): Serum inflammatory factor detection results (*n* = 5). (**F**): Serum biochemical indicator detection results (*n* = 5). ns: No significant difference. (**G**): HE-stained histological sections of heart, liver, spleen, lung, and kidney (*n* = 5).

Following the successful preparation of the *K. pneumoniae* nanovaccine candidate, we evaluated its safety profile. Balb/c mice were immunized intranasally with CNP-OPS_KpO1_ (10 μg of polysaccharide per mouse). A series of parameters were monitored over the subsequent 30 days (Figure 3A). To specifically assess the impact of the intranasal route, we first evaluated respiratory metabolism. Mice were housed in metabolic cages for 48 h post-immunization for real-time monitoring of the Heat, oxygen consumption (VO_2_), and carbon dioxide production rate (VCO_2_). No significant differences were found between the immunized and control groups (Figure 3B). Throughout the entire observation period, all mice maintained stable body temperature, and no differences in body weight changes were observed between the groups (Figure 3C,D).

Serum cytokine levels, including interleukin-6 (IL-6), interleukin-1β (IL-1β), interferon-gamma (IFN-γ), and tumor necrosis factor-alpha (TNF-α), were measured by ELISA at various time points after immunization. All cytokine levels remained relatively low, particularly within the first four days, indicating that intranasal administration of the nanoparticle did not trigger a cytokine storm (Figure 3E). Serum biochemical indices—including ALT, ALP, AST, BUN, and LDH—were measured on day 30 post-immunization and all within normal ranges (Figure 3F). Furthermore, hematoxylin and eosin (H&E) staining analysis of the lungs, liver, spleen, kidneys, and heart revealed no pathological abnormalities compared to the control group (Figure 3G and Figure S6). These collective results demonstrate that the CNP-OPS_KpO1_ nanovaccine exhibits excellent safety and biocompatibility when administered via the intranasal route.

### 3.4. Antibody Response in Mice After Nasal Immunization with CNP-OPS_KpO1_

**Figure 4:**
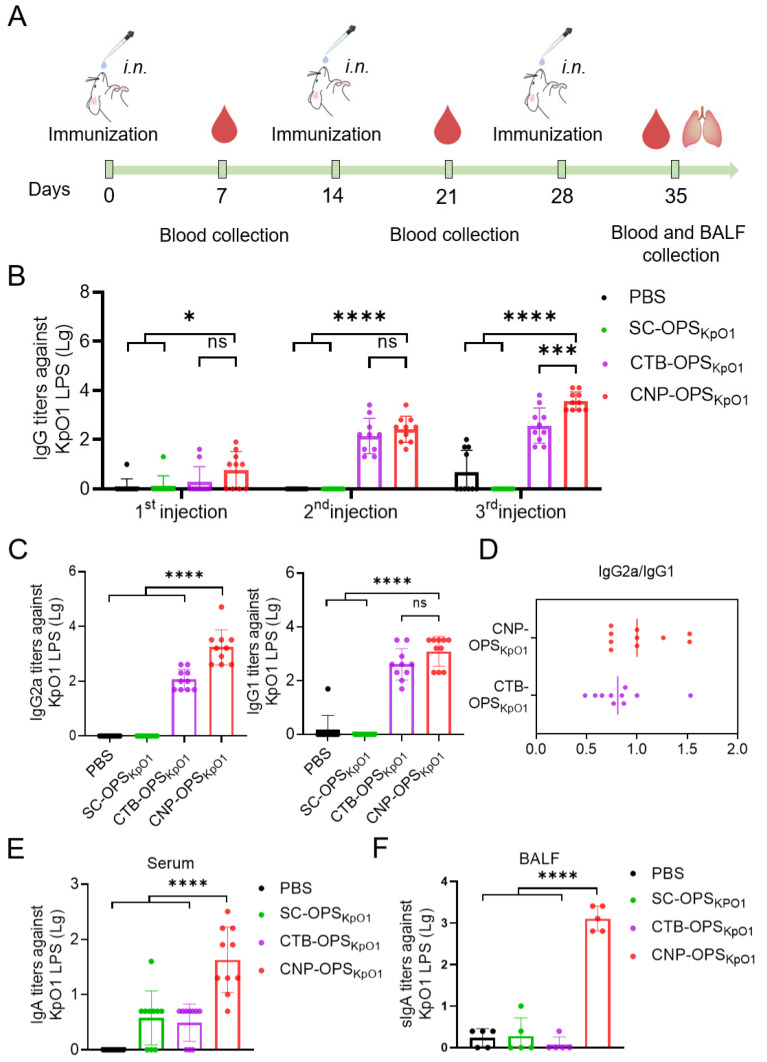
Evaluation of antibody response of CNP-OPS_KpO1_. (**A**): Schematic of the immunization design for the CNP-OPS_KpO1_. (**B**): Total IgG antibody titers against KpO1 LPS in serum after three vaccinations (*n* = 10). (**C**): Subtype IgG2a and IgG1 antibody titers after three vaccinations (*n* = 10). (**D**): Ratio of IgG2a to IgG1 after three vaccinations (*n* = 10). (**E**): IgA antibody titers in serum after three vaccinations (*n* = 10). (**F**): sIgA antibody titers in BALF after three vaccinations (*n* = 5). **** *p* < 0.0001, *** *p* < 0.001, * *p* < 0.05, ns: No significant difference.

The respiratory tract is the primary target organ for *K. pneumoniae* infection, we therefore further evaluate the efficacy of this vaccine via mucosal administration. Balb/c mice (10 per group) were immunized intranasally with PBS, SC-OPS_KpO1_, CTB-OPS_KpO1_ or CNP-OPS_KpO1_ on days 0, 14, and 28. Each vaccine dose contained 10 μg of polysaccharide. At various time points after each immunization, serum and bronchoalveolar lavage fluid (BALF) were collected to measure antibody titers against *K. pneumoniae* O1 LPS (Figure 4A). The ELISA results revealed that the polysaccharide alone (SC-OPS_KpO1_) group elicited almost no specific antibody response in serum. In contrast, despite intranasal delivery, the results demonstrated a significant increase in O1 polysaccharide-specific IgG antibody titers in the serum of mice treated with CNP-OPS_KpO1_, higher than that using CTB as a carrier (CTB-OPS_KpO1_) (Figure 4B). Subsequent analysis of serum IgG subtypes (IgG1 and IgG2a) after the third immunization showed elevated levels of both subtypes in the CNP-OPS_KpO1_ group (Figure 4C,D). In contrast, no detectable antibody titers were observed in the polysaccharide-alone (SC-OPS_KpO1_) group, suggesting both Th1 and Th2 immune response was induced by the CNP-OPS_KpO1_ formulation.

Furthermore, we detected specific IgA levels in the serum and BALF of mice after three immunizations. The results showed that serum IgA levels in the CNP-OPS_KpO1_ group were significantly higher than those in the SC-OPS_KpO1_ or CTB-OPS_KpO1_ groups (Figure 4E). More importantly, in the BALF, we found that loading OPS_KpO1_ onto our nanocarrier (the CNP-OPS_KpO1_ group) elicited a robust mucosal antibody response in mice, with IgA titers far exceeding those in the SC-OPS_KpO1_ and CTB-OPS_KpO1_ groups (Figure 4F). These results indicate that CNP is capable of effectively stimulating a potent mucosal immune response.

### 3.5. Evaluation of Vaccine Protective Efficacy Using a Pulmonary Non-Lethal Infection Model

**Figure 5:**
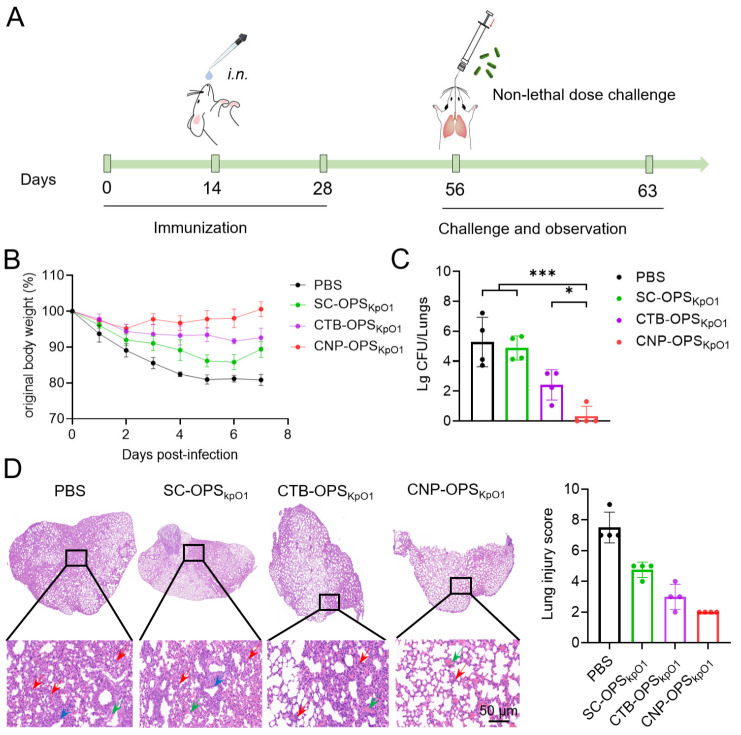
Evaluation of protective efficacy of CNP-OPS_KpO1_ in a non-lethal infection model. (**A**): Schematic of CNP-OPS_KpO1_ immunization and challenge. (**B**): Body weight changes of mice after infection with Kp041 (*n* = 4). (**C**): Pulmonary bacterial loads were measured at 36 h after infection (*n* = 4). (**D**): Representative H&E-stained images and injury scoring of lung tissue. Blue arrow: bronchial epithelial cells with loose cytoplasm and light staining; red arrow: granulocytes infiltrating the alveolar wall, accompanied by moderate-to-severe thickening of the alveolar wall and widening of the alveolar septum; green arrow: perivascular bleeding. *** *p* < 0.001, * *p* < 0.05.

To further evaluate the protective efficacy of the vaccines, we conducted a challenge via intratracheal instillation by “Micro Sprayer” at 28 days after the third immunization, using 2 × 10^3^ CFU of *K. pneumoniae* strain 041 (Kp041) per mouse. Protective indicators such as changes in body weight and bacterial load in organs were assessed post-infection (Figure 5A). Continuous monitoring of body weight revealed that mice in the PBS group experienced the most significant weight loss during the 7-day observation period, with a maximum reduction of up to 20% (Figure 5B). Although vaccination with different formulations alleviated this effect, the SC-OPS_KpO1_ and CTB-OPS_KpO1_ groups still showed maximum weight losses of approximately 15% and 10%, respectively. In contrast, the CNP-OPS_KpO1_ group performed exceptionally well, exhibiting only a slight weight loss in the first two days, followed by a rapid return to normal levels (Figure 5B).

Additionally, at 36 h post-infection, we harvested lung tissues to quantify bacterial loads. Bacterial count results showed that, compared to the PBS group (approximately 10^5^ CFU), the SC-OPS_KpO1_ group showed almost no reduction, while the CTB-OPS_KpO1_ group reduced bacterial burden by about three orders of magnitude (Figure 5C). Most notably, in the CNP-OPS_KpO1_ group, bacteria were nearly undetectable in the lungs, indicating a potent bacterial clearance capacity in vivo (Figure 5C). Furthermore, mouse lungs were collected at 36 h post-infection for H&E staining, and pathological damage was assessed. The results showed that the PBS group exhibited severe lung damage, characterized by alveolar structural destruction, inflammatory cell infiltration, and red blood cell exudation. Although the degree of damage was reduced in the SC-OPS_KpO1_ and CTB-OPS_KpO1_ groups, it remained obvious. In contrast, extremely mild tissue damage was observed in the CNP-OPS_KpO1_ group (Figure 5D).

### 3.6. Evaluation of Vaccine Protective Efficacy Using a Pulmonary Lethal Infection Model

**Figure 6:**
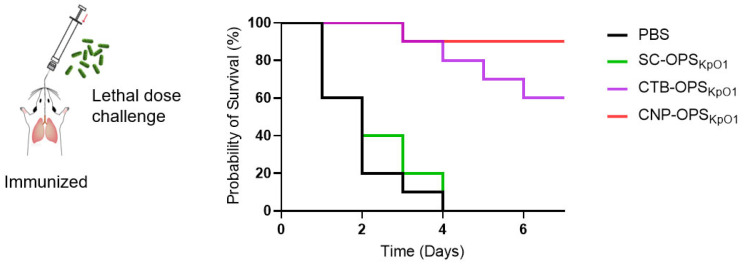
Survival curve of mice challenged with wild-type Kp041 via pulmonary delivery (*n* = 10). Mice were challenged with 9.1 × 10^4^ CFU of Kp041 per mouse via intratracheal instillation on day 7 following the final immunization, and survival of each mouse was monitored continuously.

Encouraged by the robust protective effects observed in the non-lethal challenge model, we further evaluated the candidate vaccine’s protective efficacy against a more severe, lethal dose. Mice were immunized intranasally three times as above and then challenged with a lethal dose of 9.1 × 10^4^ CFU of Kp041 per mouse via intratracheal instillation on day 7 following the final immunization. Survival was monitored continuously thereafter. During the 7-day observation period, all mice in the PBS and SC-OPS_KpO1_ groups were dead within 4 days, and in the CTB-OPS_KpO1_ groups, only 6 mice survived. In contrast, mice treated with CNP-OPS_KpO1_ showed the best protection, with 9 out of 10 mice surviving, resulting in a survival rate exceeding 90% (Figure 6). These results indicate that CNP-OPS_KpO1_ provides robust protection even against a very high, lethal pulmonary infection dose.

## 4. Discussion

In this study, we developed an innovative modular self-assembling *K. pneumoniae* bioconjugate nanovaccine (CNP-OPS_KpO1_) and evaluated its efficacy when administered via the intranasal route. In this strategy, nanocarriers and polysaccharide antigens are prepared separately and then coupled using a bioorthogonal system to enhance preparation efficiency and prevent interference between the components. The results of animal experiments indicate that this candidate can elicit robust systemic and mucosal immune responses, providing significant protection in both non-lethal and lethal pulmonary infection models without the need for adjuvants. This highlights its potential as a safe, effective, and adjuvant-free mucosal vaccine strategy against CRKP.

Mucosal immunity serves as the first line of defense at mucosal surfaces and represents a promising strategy for preventing respiratory infectious diseases. Although *K. pneumoniae* belongs to the Enterobacteriaceae family and commonly colonizes the gut, it often translocates to the lungs in immunocompromised individuals, leading to severe, difficult-to-treat infections due to its high antibiotic resistance [31]. Due to the low immunogenicity of polysaccharides themselves, a robust delivery system is required to elicit efficient immune responses. Nanoparticles offer advantages in this context, as their size and repetitive scaffold structure enable the presentation of antigens in a highly exposed conformation [22], enhancing the generation of high-titer, high-affinity specific antibodies. Additionally, the repetitive display of antigens on the particle surface promotes coaggregation of B cell receptors, which facilitates their activation. In this study, we compared the nanoparticle chassis CNP with CTB and found that the nanovaccine provide superior efficacy, suggesting a significant role for size effects. As it is known, CTB can bind to the ganglioside GM1 on the cell surface [32], which is beneficial for vaccine targeting. Notably, although CTB participated in higher-order self-assembly in our design, its GM1-binding ability was retained. This indicates that, in addition to size effects, receptor-mediated activation likely contributes to enhanced immune responses through GM1-binding-dependent mechanisms. Future studies should evaluate the contribution of this receptor effect by comparing our platform with existing nanoparticles of similar size (e.g., VLPs, ferritin nanocages) or by introducing point mutations in CNP to block GM1 binding.

Vaccine safety is an inevitable concern. The CTB module used in this study belongs to the bacterial AB_5_ toxin family [32]. In a clinical trial of an intranasal vaccine, a mutated bacterial AB_5_ toxin was used as an adjuvant, and transient facial palsy was reported in some human subjects [33]; however, such toxins typically contain both A and B subunits. In contrast, we expressed only the non-toxic B subunit through recombinant methods. In addition, studies have reported that cholera toxin (CT) can reach the olfactory bulb via the intranasal route. But there is no evidence that CTB alone causes cellular damage [34]; the major associated injuries have been attributed to the intact cholera holotoxin CT. Furthermore, in a human trial involving intranasal administration of CTB, even at a high dose (1000 µg), no neurological abnormalities were observed [35]. In this study, we preliminarily assessed the safety of the vaccine in mice and found no signs of systemic inflammatory responses, abnormalities in liver or kidney function, or respiratory or metabolic disturbances. Available data suggest that the CTB module is safe for intranasal mucosal immunization. Nonetheless, more comprehensive safety evaluations will be necessary before clinical translation.

Our results demonstrate that CNP-OPS_KpO1_ effectively elicits both systemic and mucosal immune responses. Although we have demonstrated the protective effect of this vaccine against the clinical isolate Kp041, its efficacy against additional clinical isolates requires further evaluation. It is also necessary to assess the potential advantages of this mucosal vaccination route—considering both humoral and cellular immune responses—compared to other candidate vaccines administered via different routes. Furthermore, due to the modular loading of antigen, polysaccharides from other prevalent serotypes of *K. pneumoniae* (such as O2, O3, and O5), as well as CPS and antigens from other pathogens, can theoretically be efficiently incorporated for the development of multivalent vaccines. Moreover, the recent successful fabrication of flexible, antioxidant thin films reinforced with functional nanomaterials, as reported for food packaging [36,37], inspires the possibility of developing dissolvable microneedle patches or mucosal films for this type of vaccine, which could facilitate needle-free administration.

## Data Availability

The data presented in this study are available on request from the corresponding author.

## Supplementary Materials

The following supporting information can be downloaded at https://www.mdpi.com/article/10.3390/nano16070428/s1. Figure S1. DLS analysis of CNP (Intensity). Figure S2: SC-OPS_KpO1_ Ion Exchange Column (Q column) Purification Coomassie Blue Staining Pattern. (Sample 0: Sample applied before Q column; Sample 1–26: Samples eluted sequentially by gradient on Q column.). Figure S3. Integrated Optical Density of the CNP band in the gel shown in Figure 1E. Figure S4. CNP-OPS_KpO1_ Molecular Sieve Coomassie Blue Staining Pattern. (CNP + OPS_KpO1_: Sample prior to loading onto the molecular sieve; CNP-OPS_KpO1_ and SC-OPS_KpO1_: Separated samples.). Figure S5. DLS analysis of CNP-OPS_KpO1_ (Intensity). Figure S6. Representative images of H&E staining of tissue sections from the heart, liver, spleen, lungs, and kidneys of CNP-OPS_KpO1_-immunized mice at both high and low magnifications.
