# Supplementary material for: Modular Development of a Klebsiella pneumoniae Bioconjugate Nanovaccine Elicits Robust Protection via Intranasal Immunization
Source: Nanomaterials (Basel). 2026 Mar 31;16(7):428. doi: 10.3390/nano16070428 (PMC13074939; doi:10.3390/nano16070428)
Supplement: Supplementary file 1 [file nanomaterials-16-00428-s001.zip › nanomaterials-4175223-supplementary.pdf]

Supporting information

Modular Development of a *Klebsiella pneumoniae* Bioconjugate Nanovaccine Elicits Robust Protection via Intranasal Immunization

Zhenshi Li [1,2], Lingli Chen [2,3], Canran Liu [2,3], Kangfeng Wang [2], Juntao LI [2], Xue Yan [2], Yuqing Jiang [2], Yan Guo [2], Li Zhu [2], Hengliang Wang [1,4,*], Chao Pan [2,*]

[1] College of Food Science and Technology, Shanghai Ocean University, Shanghai 201306, China; 18716532473@163.com (Z.L.)

[2] National Key Laboratory of Advanced Biotechnology, Academy of Military Medical Science, Beijing 100071, China; wangkf1220@126.com(K.W.) ; ljtanlz0046@163.com(J.L.); Cynthia2182@163.com(X.Y.); jyq1214179706@163.com(Y.J.); yanguobrilliant@163.com (Y.G.) ; jewly54@bmi.ac.cn (L.Z.)

[3] School of Basic Medical Sciences, Fujian Medical University, Fuzhou, Fujian 350108, China；Chenlinglizy@163.com(L.C.); 18772103166@163.com(C.L.)

[4] State Key Laboratory of Pathogen and Biosecurity, Academy of Military Medical Sciences, Beijing 100071, China.

* Correspondence: panchao@bmi.ac.cn (C.P.); wanghl@bmi.ac.cn (H.W.)

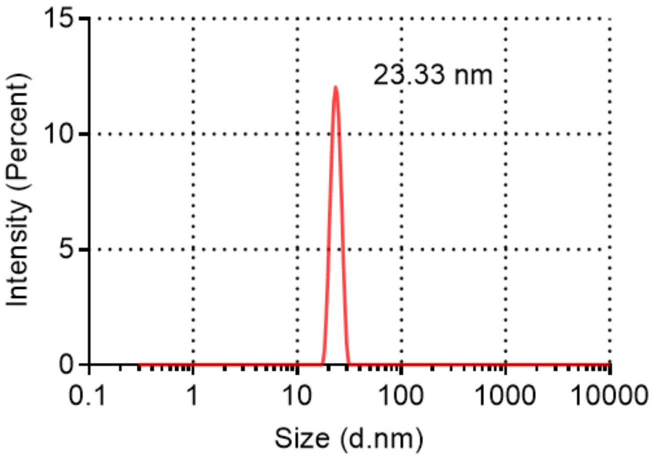

Figure S1. DLS analysis of CNP (Intensity).

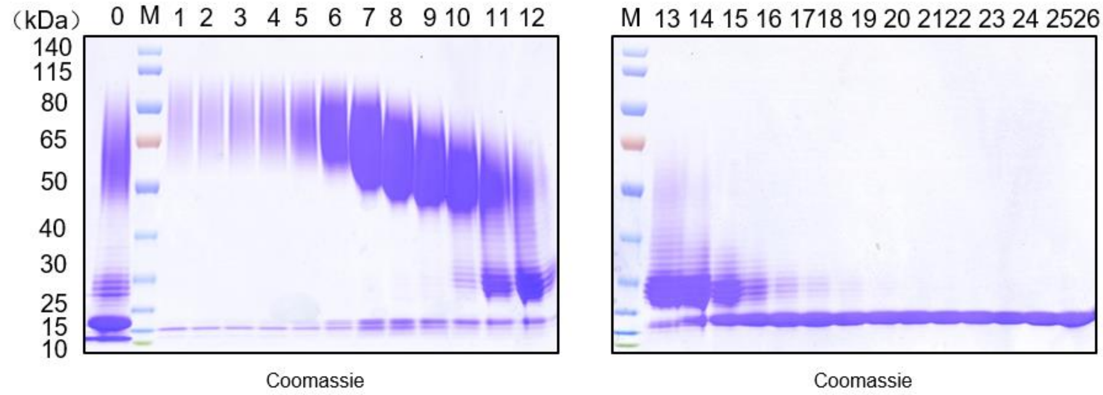

Figure S2. SC-OPS$_{KpO1}$ Ion Exchange Column (Q column) Purification Coomassie Blue Staining Pattern. (Sample 0: Sample applied before Q column; Sample 1–26: Samples eluted sequentially by gradient on Q column.)

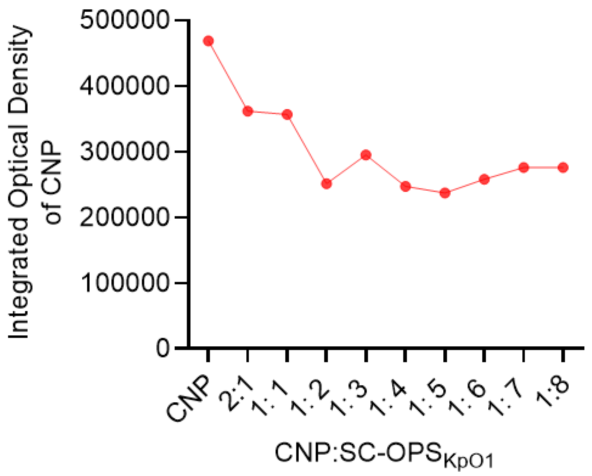

Figure S3. Integrated Optical Density of the CNP band in the gel shown in Fig. 1E.

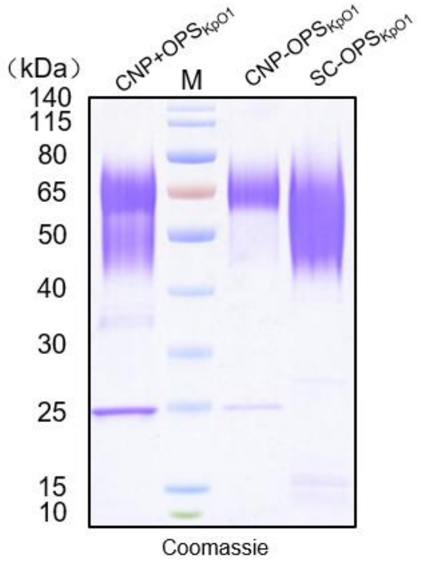

Figure S4. CNP-OPS$_{KpO1}$ Molecular Sieve Coomassie Blue Staining Pattern. (CNP+OPS$_{KpO1}$: Sample prior to loading onto the molecular sieve; CNP-OPS$_{KpO1}$ and SC-OPS$_{KpO1}$: Separated samples.)

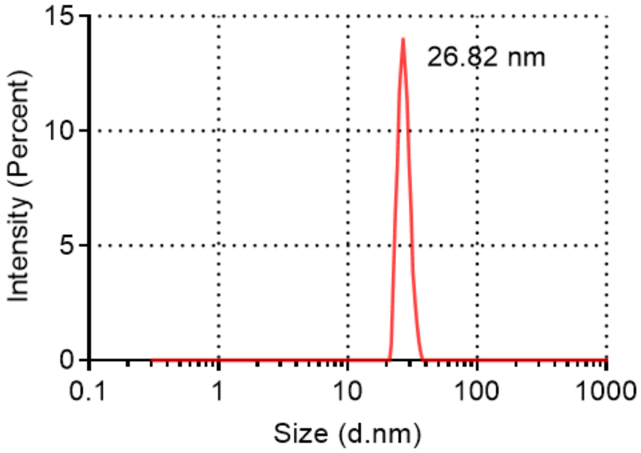

Figure S5. DLS analysis of CNP-OPS$_{KpO1}$ (Intensity).

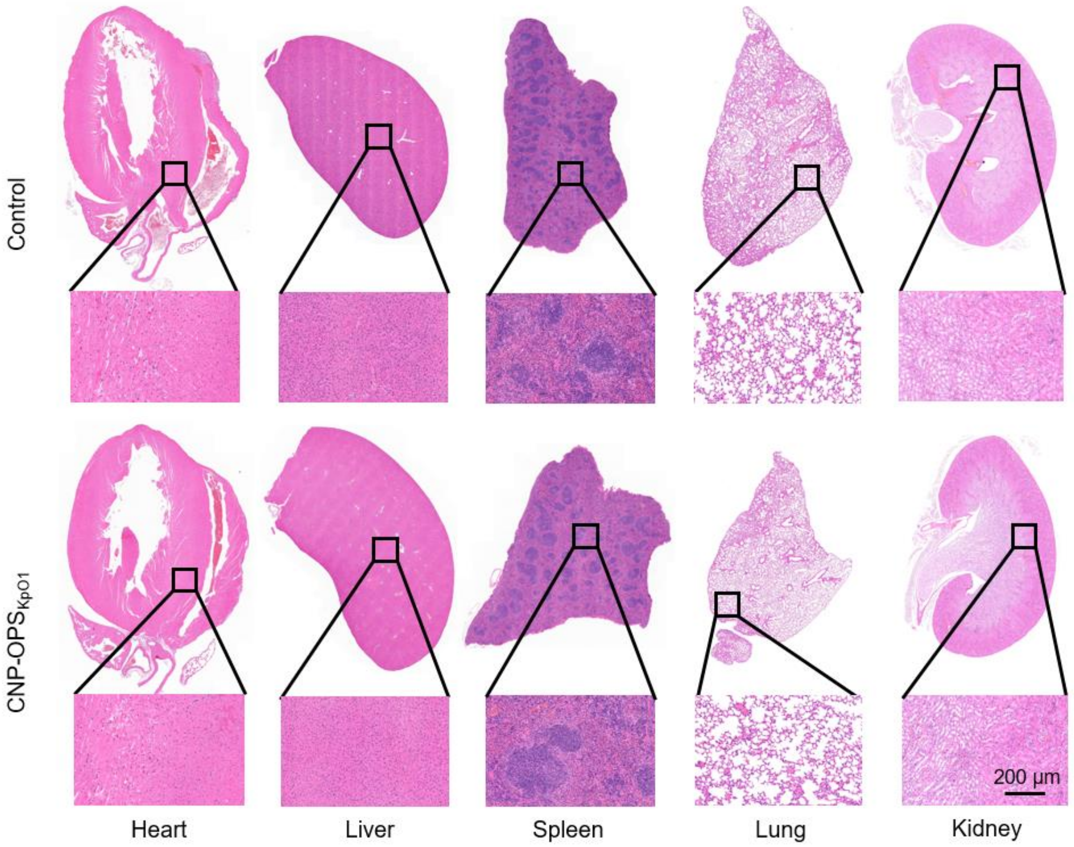

Figure S6. Representative images of H&E staining of tissue sections from the heart, liver, spleen, lungs, and kidneys of CNP-OPS$_{KpO1}$-immunized mice at both high and low magnifications.
